# Whole-brain volumetric analysis in adult Moyamoya patients reveals significant atrophy compared to healthy controls

**DOI:** 10.1093/braincomms/fcaf100

**Published:** 2025-03-03

**Authors:** Patrick Haas, Alexander Debolski, Benjamin Bender, Leonie Zerweck, Ulrike Ernemann, Marcos Tatagiba, Till-Karsten Hauser, Nadia Khan, Constantin Roder

**Affiliations:** Department of Neurosurgery and Moyamoya Center, University of Tübingen, Tübingen 72076, Germany; Department of Neurosurgery and Moyamoya Center, University of Tübingen, Tübingen 72076, Germany; Department of Neuroradiology, University of Tübingen, Tübingen 72076, Germany; Department of Neuroradiology, University of Tübingen, Tübingen 72076, Germany; Department of Neuroradiology, University of Tübingen, Tübingen 72076, Germany; Department of Neurosurgery and Moyamoya Center, University of Tübingen, Tübingen 72076, Germany; Department of Neuroradiology, University of Tübingen, Tübingen 72076, Germany; Department of Neurosurgery and Moyamoya Center, University of Tübingen, Tübingen 72076, Germany; Moyamoya Center, University Children’s Hospital Zurich, Zurich 8032, Switzerland; Department of Neurosurgery and Moyamoya Center, University of Tübingen, Tübingen 72076, Germany

**Keywords:** cerebral revascularization, Moyamoya, volumetric, healthy controls

## Abstract

Moyamoya disease (MMD) may lead to perfusion deficits, stroke and brain atrophy in the long-term. Our aim was to analyse whole-brain volumetry of a large cohort of Moyamoya disease patients compared to healthy controls. 3D T1w MRI sequences of adult Moyamoya disease patients treated at our centre between 2016 and 2022 without prior revascularization were analysed for whole-brain volumetry (AssemblyNet) and compared age-controlled to healthy controls. A total of 133 different regions of interest were examined retrospectively for each patient separately by localization, structure and tissue type. All segmentations were subjected to automated and manual quality control. After quality control, 149 hemispheres from 80 Moyamoya disease patients were compared to 258 hemispheres from 129 healthy controls. A significant brain volume loss was observed in Moyamoya disease patients with increasing age, with the greatest reduction seen in bilaterally affected patients with Suzuki grade >3. As direct signs of brain atrophy, significant differences were seen across all regions of interests, emphasized in cortical grey matter with a reduction of 4.4% (95% CI 2.7–6.1%; *P* < 0.001) in patients aged 30–45 years and 3.4% (95% CI 2.1–4.7%; *P* < 0.001) aged 46–60 years. As indirect sign for atrophy, external CSF spaces increased up to 26.4% (95% CI 17.0–35.9%; *P* < 0.001) for 30–45 years and 28.4% (95% CI 17.1–39.7%; *P* < 0.001) for 46–60 years compared to healthy controls. Infratentorial, significant volume loss was observed for patients aged 46–60 years with 11.6% for cerebellar white matter (95% CI 3.7–19.5%; *P* = 0.0025) and with 8.5% (95% CI 3.5–13.5%; *P* = 0.0006) for the brainstem, likely due to secondary neurodegeneration. Moyamoya disease patients >45 year without ischaemia also had significantly less grey matter and white matter volume, with accordingly enlarged CSF spaces. Moyamoya disease may lead to significant differences in brain volume of local and global regions of interest as a sign of brain atrophy, even in the absence of infarctions. These findings might be useful for the understanding of the disease burden and in decision-making for timely revascularization.

## Teaser

Haas *et al*. compared the brain volumes of Moyamoya patients with those of healthy individuals. They revealed a previously undocumented percentage difference of brain atrophy depending on the severity of the disease, showing that Moyamoya can affect the entire brain even without a manifest stroke.

## Graphical Abstract

**Graphical Abstract:**
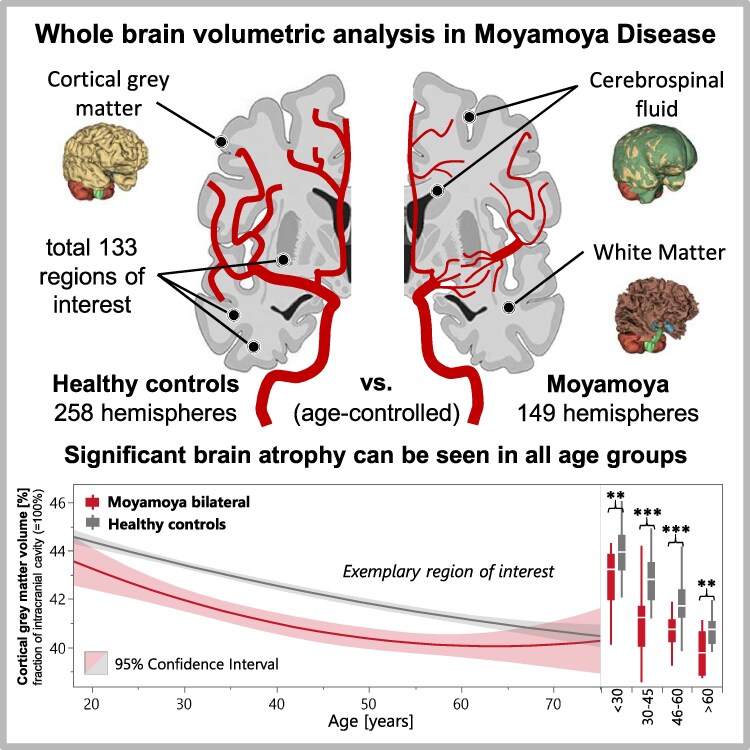

## Introduction

Moyamoya disease (MMD) is a steno-occlusive angiopathy of the terminal parts of the internal carotid artery of unclear pathogenesis, leading to collateral vessel formation bypassing the affected vascular segments of the circle of Willis, which appear angiographically as a rete mirabile.^1,2^ However, these collaterals may be at high risk of acute and/or chronic cerebrovascular decompensation leading to recurrent transient ischaemic attacks (TIAs) and haemorrhagic or ischaemic strokes on one hand, and brain atrophy due to chronic hypoperfusion. As the disease is most likely to develop in children and young adults, the lifetime risk for developing neurological and neuropsychological deficits is high.^3,4^ To date, little is known about whether such prolonged chronic hypoperfusion of the brain, even in the absence of obvious infarcts, can lead to structural changes, seen as brain atrophy. Therefore, the aim of this study was to analyse whole-brain volumetric (WBV) of a large European MMD cohort compared to healthy controls and to correlate the findings with other imaging modalities, as well as clinical data.

## Materials and methods

### Inclusion and exclusion criteria

All adult MMD patients aged 16–80 years treated at our centre from June 2016–June 2022 without prior revascularization and available high-resolution (slice thickness ≤1 mm) 3D T1w MRI sequences were considered for retrospective WBV analysis. The 3D MPRAGE sequences were measured at a 3T Skyra (Siemens, Erlangen) (1.0 × 1.0 × 1.0 mm^3^ repetition time (TR)/echo time (TE)/inversion time (TI) 2300/2.3/900 ms, flip angle 8°) until May 2021 there was a machine update on a 3T Vida (Siemens, Erlangen) (1.0 × 1.0 × 1.0 mm^3^ TR/TE/TI 2300/3.5/1100 ms, flip angle 8°). Diagnosis and disease classification were done according to the 2021 Japanese guidelines for MMD.^5^ Angiographic disease stage was categorized according to Suzuki, while white matter (WM) lesions with T2-flair hyperintensities were categorized into Fazekas grades.^2,6^ Anonymized MRI datasets from healthy subjects between 18 and 85 years of age, acquired between 2012 and 2016 were used as a reference dataset. Inclusion criteria and measurement parameter have been described previously: in short the 3D MPRAGE (0.9 × 0.9 × 0.9 mm^3^, TR/TE/TI 2300/2.3/900 ms, flip angle 8°, GRAPPA = 2) was measured at a 3T Skyra (Siemens, Erlangen).^7^

### Data processing, workflow and statistics

**Figure 1:**
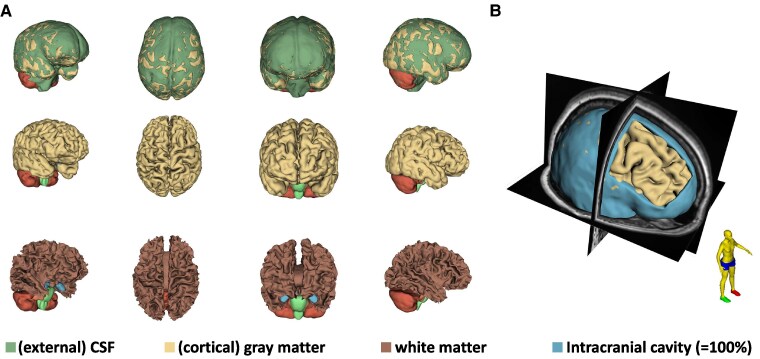
**3D visualizations of the WBV.** (**A**) Volumes of exCSF (green), cGM (beige) and WM (dark brown). (**B**) Representation of the intracranial cavity (blue) in spatial orientation, which serves as an inter-individual reference for the ROIs studied.

All 3D T1w MRI sequences were pseudonymously extracted from the picture archiving and communication system (PACS). Subsequently, further anonymization was performed with removal of all patient-specific features by defacing (PyDeface v2.0.2). Data processing for WBV was performed by AssemblyNet, which provides volumetric results of 133 labels based on convolutional neural networks (CNN).^8^ These distinct regions of interests (ROIs) included side-separated parenchymal macrostructures of all lobes and gyri, with subdivisions as WM and grey matter (GM), cortical and subcortical structures and CSF space (Fig. 1A). Any calculated absolute values were normalized to the intracranial cavity (=100%) for interindividual comparability (Fig. 1B). At the end of the processing pipeline, an objective rating was performed using CNN-based quality control (RegQCNET) with a three-level rating scale (A–C) to verify the segmentation results.^9^ Data from the best category A were accepted, data from category B were subjected to further manual QC while results from category C were excluded. Server-based REDCap database software (Version 9.6.1) was used to store patient data. Data analysis and statistics were performed in JMP (JMP 16.0, SAS Institute). Visualizations and 3D rendering were created using 3D Slicer (Version 4.13.0).^10^ The alpha level was defined as.05. Variance homogeneity was tested with Levene’s test and pooled *t*-test or Welch’s test was applied accordingly. To avoid alpha error accumulation in multiple testing, *P*-values were adjusted according to the Bonferroni–Holm correction. A one-way ANOVA was performed on the data from multiple groups, followed by a Tukey-Kramer *post hoc* test. In the case of parametric data, the 95% CI was additionally reported. Cohen’s *d* and *d_z_* were calculated as measures of effect size for unpaired and paired *t*-tests, respectively. Polynomial of *n*th degree was used as regression curves of volumes as a function of age. The highest coefficient of determination *R*^2^ was obtained for *n* = 2, so second degree polynomials were used.

### Standard protocol approvals, registrations and patient consents

All procedures performed in studies involving human participants were conducted in accordance with the ethical standards of the Institutional Research Committee and the 1964 Helsinki Declaration and its later amendments or comparable ethics standards. Approval of the Ethics Committee of the University Hospital Tübingen was granted (937/2021BO2). Due to the retrospective character of this analysis no specific formal consent of the participating patients was obtained. Statistical advice was provided by the Institute for Clinical Epidemiology and Applied Biometry of the authors’ institute.

## Results

### Healthy controls

The healthy control group (HC) consisted of *n* = 258 examined hemispheres from *n* = 129 subjects with *n* = 64 male (49.6%) and *n* = 65 female (50.4%) subjects with a balanced age distribution [median 36 years (19–85); mean 39.4 ± 15.5]. After QC of all segmented hemispheres, there were no data drop-outs. All subjects had a normal neurological examination and normal cognition (tested with DemTect).^7^

### MMD study cohort

**Figure 2:**
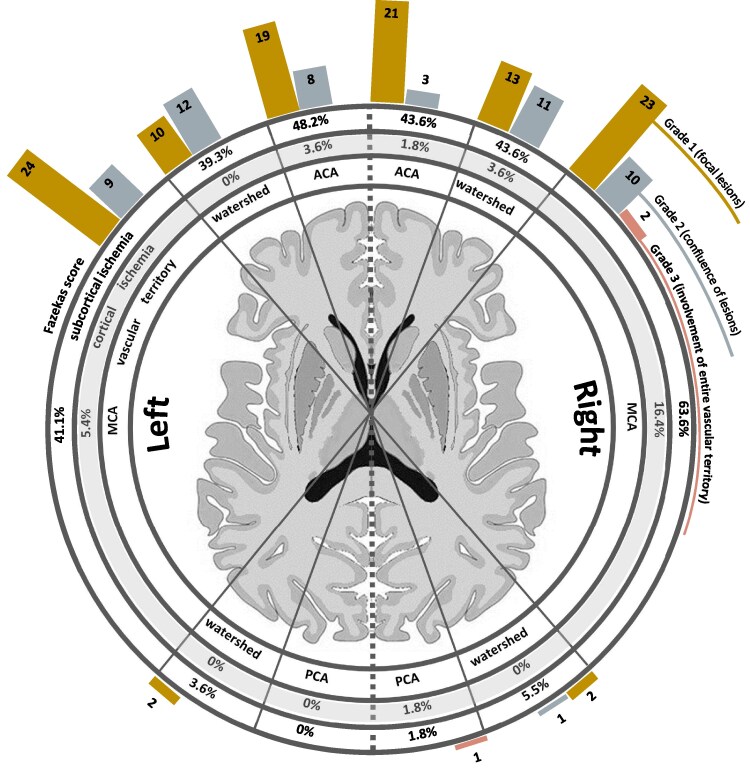
**Distribution of cortical and subcortical ischaemia.** Percentage distribution of cortical (middle ring) and subcortical ischaemia (outer ring) with subdivision according to Fazekas (modified bar chart) into focal lesions, confluence of lesions, involvement of entire vascular territory according to vascular territories [ACA, MCA and posterior cerebral artery (PCA)] and in-between watersheds (inner ring) for the MMD study cohort (*n* = 80 individual patients).

The MMD cohort studied consisted of *n* = 164 hemispheres from *n* = 82 patients. After QC, *n* = 149 hemispheres of *n* = 80 individual MMD patients remained for further analysis (female-to-male ratio of 2.5:1 (*n* = 57 female, *n* = 23 male); median 41 years (16–75), mean 43.2 years (±13.4). Of the 149 hemispheres analysed, *n* = 113 were directly affected by MMD, the remaining *n* = 36 were non-affected hemispheres of unilateral MMD (uniMMD) patients. The distribution of hemispheric affection was homogeneous: 54.4% had bilateral (biMMD) and 45.6% uniMMD. Digital subtraction angiography for assessment of Suzuki grading was available in *n* = 79 patients (98.8%). Suzuki Grade 3 was the most common disease stage. Signs of cortical and/or subcortical ischaemia in terms of T2-FLAIR hyperintensities on MRI were seen in 80 of the 111 MMD-affected hemispheres (72.1%), whereas 31 MMD-affected hemispheres (27.9%) showed no signs of ischaemia. The detailed distribution and intensity of ischaemic subcortical WM lesions (Fazekas score, as described previously 11) can be found in Table 1 and Fig. 2. No cases of infratentorial ischaemia or lesion were identified in any of the patients.

**Table 1:** Patient characteristics

Cohort	MMD	HC
Total (individuals/hemispheres)		
Screening	82/164	129/258
Analysis after QC	80/149	129/258
MMD-affected	80/113	
Sex [individuals (%)]	80 (100%)	129 (100%)
Female	57 (71.3%)	65 (50.4%)
Male	23 (28.7%)	64 (49.6%)
Age (years ± SD)	43.2 ± 13.4	39.4 ± 15.5
MMD type [individuals (%)]	80 (100%)	
Unilateral	36 (45.0%)	
Right	17 (21.3%)	
Left	19 (23.8%)	
Bilateral	44 (55.0%)	
Suzuki score [hemispheres (%)]^a^		
Grade 1	9 (8.0%)	
Grade 2	14 (12.5%)	
Grade 3	43 (38.4%)	
Grade 4	21 (18.7%)	
Grade 5	19 (17.0%)	
Grade 6	6 (5.4%)	
MMD onset	80 (100%)	
Incidental	3 (3.8%)	
Minor performance deficit	13 (16.3%)	
transient ischaemic attack (TIA)	22 (27.5%)	
Ischaemic stroke	37 (46.3%)	
intracerebral hemorrhage (ICH)	5 (6.3%)	

^a^One DSA was not available.

### Volumetric comparison of the MMD and HC cohorts—supratentorial and CSF spaces

**Figure 3:**
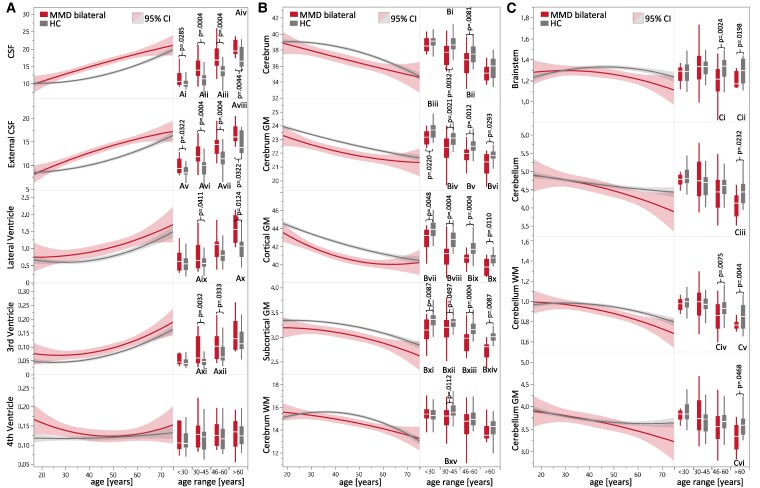
**Brain volume differences between biMMD and healthy controls.** (**A–C**) Volumes of the MMD collective (red) and the HC (dark grey). Left side: Curve plot of ROI volumes (percentage in relation to intracranial cavity) versus patient age in years as second degree polynomials (highest coefficient of determination *R*^2^). The associated 95% CI is shaded. Significant differences can be assumed in the range of non-overlapping confidence intervals. Right side: Boxplots of ROI volumes split into age groups <30 (*n* = 112), 30–45 (*n* = 92), 46–60 (*n* = 88) and >60 years (*n* = 46). The *t* statistics of the *t*-tests performed are Ai: *t*(16.6) = −2.04, Aii: *t*(33.2) = −4.9, Aiii: *t*(36.4) = −4.94, Aiv: *t*(19.1) = −3.23, Av: *t*(17.8) = −2.27, Avi: *t*(38.8) = −5.09, Avii: *t*(36.9) = −5.1, Aviii: *t*(15) = −2.36, Aix: *t*(26,7) = −2.33, Ax: *t*(17.9) = −3.1, Axi: *t*(27.5) = −3.5, Axii: *t*(46.1) = −2.37, Bi: *t*(28.4) = 3.47, Bii: *t*(35.8) = 2.96, Biii: *t*(16.1) = 2.54, Biv: *t*(28) = 3.54, Bv: *t*(36.1) = 3.79, Bvi: *t*(11.4) = 2.1, Bvii *t*(17.6) = 3.22, Bviii: *t*(31.9) = 5.32, Bix: *t*(41.2) = 4.81, Bx: *t*(16.1) = 2.54, Bxi: *t*(16.7) = 3.17, Bxii: *t*(27.6) = 1.71, Bxiii: *t*(32.5) = 4.1, Bxiv: *t*(13.9) = 3.19, Bxv: *t*(30.8) = 2.98, Ci: *t*(36.1) = 3.51, Cii: *t*(22.2) = 2.7, Ciii: *t*(14) = 2.9, Civ: *t*(33.3) = 3.01, Cv: *t*(38.7) = 3.28, Cvi: *t*(12.3) = 2.58. *P*-values are adjusted according to Bonferroni–Holm correction (14 × 4). (**A**) CSF spaces. (**B**) Supratentorial ROIs (selection). (**C**) Infratentorial ROIs (selection).

A significant loss of supratentorial brain volume was observed in the MMD group across all age groups (Fig. 3B). This was true not only for the total volume of the cerebrum, but also for the subdivision into the macrostructures WM and GM, the latter in turn subdivided into the cortical (cGM) and subcortical (scGM) portions. The volume differences were most pronounced in the GM and its cortical part. In the age groups 30–45 and 46–60 years with biMMD, there was a 4.4% (95% CI 2.7–6.1%; *P* < 0.001; *d* = 1.62) and 3.4% (95% CI 2.1–4.7%; *P* < 0.001; *d* = 1.23) reduction in cGM, respectively, compared to HC (Fig. 3B). In further analysis of individual areas of WM, cGM and scGM, the putamen, pallidum, caudate, frontal lobe with middle frontal gyrus (MFG) and precentral gyrus (PrG), as well as insula with anterior (AIns) and posterior (PIns) portions and adjacent planum polare (PP) as part of the superior temporal gyrus (STG) showed significant volume deficits compared to the corresponding healthy age cohort (Tables 2 and 3).

**Table 2:** Volume differences of 64 examined ROIs without macrostructures between MMD and HC subdivided by 4 age cohorts

ROI	Δ Volume (%) (95% CI)		*P*-value adjusted	Effect size *d*
	Age range (years)	<30		
		30–45		
		46–60		
		>60		
Accumbens	8.4%	(2.1–14.8%)	0.255	
	7%	(0.1–13.9%)	>0.999	
	9.1%	(1.8–16.3%)	0.4234	
	0.1%	(−13.4–13.5%)	>0.999	
Amygdala	−1.2%	(−4.9–2.6%)	>0.999	
	0.9%	(−2.4–4.2%)	>0.999	
	1%	(−2.9–5%)	>0.999	
	0.9%	(−5.7–7.6%)	>0.999	
Basal Forebrain	9.4%	(3.5–5.3%)	0.057	
	−3%	(−9.1–3.2%)	>0.999	
	2.3%	(−2.9–7.5%)	>0.999	
	2.7%	(−7.9–13.3%)	>0.999	
Caudate	11%	(6.2–15.8%)	0.0064**	0.59
	3.9%	(−1.4–9.2%)	>.999	
	9%	(1.5–16.4%)	0.5208	
	19.4%	(10.9–27.9%)	0.0064**	0.87
Hippocampus	0.8%	(−3.2–4.8%)	>0.999	
	−4.2%	(−8.2–−0.2%)	>0.999	
	0.5%	(−2.8–3.8%)	>0.999	
	−8.1%	(−14–−2.1%)	>0.999	
Pallidum	3.2%	(−0.6–7%)	>0.999	
	−1.1%	(−5.8–3.7%)	>0.999	
	5.9%	(1.1–10.6%)	0.4446	
	15.8%	(7.7–24%)	0.0064**	0.87
Putamen	4.7%	(0.9–8.5%)	0.4	
	2.7%	(−1.8–7.1%)	>0.999	
	6.1%	(1.5–10.7%)	0.2714	
	16.2%	(8.5–24%)	0.0064**	0.87
Thalamus	4.2%	(1.5–6.8%)	0.0715	
	3.2%	(0–6.3%)	>0.999	
	2.9%	(0.2–5.6%)	0.915	
	4.8%	(−0.8–10.4%)	>0.999	
Ventral DC	−0.7%	(−3.5–2.2%)	>0.999	
	−4.6%	(−7.7–−1.5%)	>0.999	
	2.3%	(−0.5–5.1%)	>0.999	
	4.7%	(0.3–9.1%)	>0.999	
Frontal	5.2%	(3.3–7.2%)	0.0064**	0.59
	5.6%	(3.4–7.7%)	0.0064**	0.52
	3.8%	(1.8–5.9%)	0.0128*	0.47
	3.1%	(0.3–5.8%)	>0.999	
Fronto-polar gyrus (FRP)	3.2%	(−2.1–8.6%)	>0.999	
	6.6%	(1.7–11.5%)	0.2279	
	−0.4%	(−4.5–3.6%)	>0.999	
	−2.6%	(−10–4.7%)	>0.999	
Gyrus rectus (GRe)	2.9%	(−4.4–10.2%)	>0.999	
	0.8%	(−6.7–8.3%)	>0.999	
	1%	(−5.5–7.5%)	>0.999	
	−2.1%	(−12.2–8%)	>0.999	
Inferior frontal gyrus opercular part (OpIFG)	3.1%	(−5.9–12.2%)	>0.999	
	6.3%	(−1.8–14.4%)	>0.999	
	8.5%	(−0.3–17.3%)	>0.999	
	9.5%	(−3.6–22.5%)	>0.999	
Inferior frontal gyrus orbital part (OrIFG)	10.6%	(−0.8–22%)	>0.999	
	4%	(−5.6–13.6%)	>0.999	
	−1.7%	(−11–7.5%)	>0.999	
	5.2%	(−10.2–20.5%)	>0.999	
Inferior frontal gyrus triangular (TrIFG)	6.6%	(−2.7–15.9%)	>0.999	
	1.9%	(−8.1–11.8%)	>0.999	
	−4.7%	(−13.2–3.8%)	>0.999	
	0.7%	(−14.5–16%)	>0.999	
Medial frontal cortex (MFC)	−1.6%	(−10.2–6.9%)	>0.999	
	4.2%	(−3.8–12.2%)	>0.999	
	1%	(−6.8–8.7%)	>0.999	
	−1.1%	(−14.7–12.5%)	>0.999	
Middle frontal gyrus (MFG)	5.5%	(1.4–9.6%)	0.2444	
	8.3%	(3.8–12.9%)	0.0118*	0.48
	7.7%	(3.5–11.9%)	0.0128*	0.47
	7.2%	(1.7–12.7%)	0.3712	
Orbital gyrus anterior part (AOrG)	−3.2%	(−13.3–6.9%)	>0.999	
	0.2%	(−8.4–8.7%)	>0.999	
	3.2%	(−5–11.4%)	>0.999	
	0.8%	(−11.3–13%)	>0.999	
Orbital gyrus lateral part (LOrG)	3.4%	(−5.2–11.9%)	>0.999	
	0.7%	(−7.1–8.5%)	>0.999	
	−6.6%	(−14.2–1%)	>0.999	
	−1%	(−12.2–10.3%)	>0.999	
Orbital gyrus medial part (MOrG)	−3.6%	(−9.3–2%)	>0.999	
	0.3%	(−4.3–4.9%)	>0.999	
	0.9%	(−3.4–5.2%)	>0.999	
	−6.3%	(−12.6–0%)	>0.999	
Orbital gyrus posterior part (POrG)	3.5%	(−3.6–10.5%)	>0.999	
	0.1%	(−5.5–5.7%)	>0.999	
	−2.3%	(−7.8–3.2%)	>0.999	
	2.7%	(−6.4–11.9%)	>0.999	
Precentral gyrus (PrG)	8.1%	(4.2–11.9%)	0.0064**	0.59
	9.1%	(5.2–13%)	0.0064**	0.52
	3.5%	(0.5–6.4%)	0.605	
	8.1%	(2.1–14%)	0.288	
PRG medial segment (MPrG)	4.5%	(−2.2–11.2%)	>0.999	
	4.6%	(−1.4–10.6%)	>0.999	
	5.1%	(−0.8–11.1%)	>0.999	
	1.3%	(−9.2–11.9%)	>0.999	
Subcallosal area (SCA)	19.3%	(7.2–31.5%)	0.057	
	14.1%	(4.2–24.1%)	0.1595	
	3.2%	(−5–11.3%)	>0.999	
	−6.5%	(−22.3–9.3%)	>0.999	
Superior frontal gyrus (SFG)	6.5%	(2.3–10.7%)	0.081	
	6.2%	(1.8–10.6%)	0.162	
	4.1%	(−0.5–8.8%)	>0.999	
	−1.2%	(−8.2–5.8%)	>0.999	
SFG medial part (MSFG)	6.2%	(−0.6–13%)	>0.999	
	1.4%	(−4.8–7.6%)	>0.999	
	6.1%	(−0.8–13.1%)	>0.999	
	3.1%	(−7.6–13.8%)	>0.999	
Supplementary motor cortex (SMC)	6.2%	(0.3–12.2%)	0.9898	
	4.5%	(−0.4–9.5%)	>0.999	
	4.7%	(−0.3–9.8%)	>0.999	
	4.4%	(−3.1–11.9%)	>0.999	
Temporal	1%	(−1.4–3.4%)	>0.999	
	1.5%	(−1.1–4.1%)	>0.999	
	0.7%	(−1.3–2.7%)	>0.999	
	−1.4%	(−5.4–2.7%)	>0.999	
Fusiform gyrus (FuG)	2.6%	(−2.7–7.9%)	>0.999	
	0.2%	(−4.8–5.2%)	>0.999	
	−2.1%	(−7.6–3.4%)	>0.999	
	−1%	(−11–8.9%)	>0.999	
Planum polare (PP)	9.2%	(3.6–14.8%)	0.0464*	0.40
	3.2%	(−2.9–9.3%)	>0.999	
	4%	(−1.1–9%)	>0.999	
	−1.6%	(−10–6.8%)	>0.999	
Planum temporale (PT)	−3.3%	(−14.3–7.7%)	>0.999	
	4.9%	(−5.4–15.2%)	>0.999	
	−1.3%	(−10.8–8.2%)	>0.999	
	2.2%	(−14.2–18.7%)	>0.999	

Positive Δ volume values correspond to volume decreases in MMD. *P*-values adjusted according to Bonferroni–Holm across all ROIs per age group (64 × 4). Effect size: Cohen’s *d*.

^*^ *P* < 0.05.

^**^ *P* < 0.01.

**Table 3:** Further volume differences between MMD and HC, as described in Table 2

ROI	Δ Volume (%) (95% CI)		*P*-value adjusted	Effect size *d*
	Age range (years)	<30		
		30–45		
		46–60		
		>60		
Inferior temporal gyrus (ITG)	1.5%	(−2.9–5.9%)	>0.999	
	−0.6%	(−5.3–4.1%)	>0.999	
	3%	(−0.9–6.9%)	>0.999	
	−2.9%	(−10.2–4.4%)	>0.999	
Middle temporal gyrus (MTG)	1.5%	(−3–6.1%)	>0.999	
	1.2%	(−3–5.4%)	>0.999	
	5.6%	(1.6–9.7%)	0.2074	
	2.3%	(−4.9–9.5%)	>0.999	
Superior temporal gyrus (STG)	−0.2%	(−5.5–5.2%)	>0.999	
	5.9%	(0.4–11.4%)	0.9016	
	0%	(−5.5–5.5%)	>0.999	
	−2.3%	(−10.3–5.7%)	>0.999	
Transverse temporal gyrus (TTG)	5.9%	(−2.8–14.7%)	>0.999	
	4.7%	(−3.7–13%)	>0.999	
	−5.5%	(−13.9–2.9%)	>0.999	
	2.8%	(−13.6–19.2%)	>0.999	
Temporal pole (TMP)	−2.6%	(−7.7–2.4%)	>0.999	
	0.7%	(−4.4–5.7%)	>0.999	
	−7%	(−11.6–−2.3%)	>0.999	
	−6.6%	(−16.4–3.2%)	>0.999	
Parietal	1.5%	(−1–4%)	>0.999	
	4.5%	(1.6–7,3%)	0.0754	
	3.3%	(0.9–5.7%)	0.252	
	3.3%	(−0.6–7.2%)	>0.999	
Angular gyrus (AnG)	3.9%	(−3.4–11.3%)	>0.999	
	5.4%	(−1.3–12.2%)	>0.999	
	12.2%	(5.6–18.8%)	0.0128*	0.47
	8.1%	(−4.1–20.4%)	>0.999	
Postcentral gyrus (PoG)	3.2%	(−1.3–7.7%)	>0.999	
	6.3%	(1–11.5%)	0.4998	
	4.4%	(0.6–8.2%)	0.6448	
	5.9%	(−0.3–12.2%)	>0.999	
PoG medial segment (MPoG)	−10.3%	(−20.7–0.1%)	>0.999	
	2.6%	(−7.2–12.3%)	>0.999	
	−1.4%	(−11–8.2%)	>0.999	
	−6.9%	(−25–11.3%)	>0.999	
Precuneus (PCu)	−0.9%	(−4.4–2.7%)	>0.999	
	1.3%	(−2.3–4.8%)	>0.999	
	−0.1%	(−3.8–3.6%)	>0.999	
	−0.1%	(−6.3–6%)	>0.999	
Superior parietal lobule (SPL)	0%	(−5.2–5.3%)	>0.999	
	1.2%	(−4–6.3%)	>0.999	
	−3.9%	(−8.2–0.3%)	>0.999	
	0.8%	(−6.5–8%)	>0.999	
Supra-marginal gyrus (SMG)	2.6%	(−2.7–8%)	>0.999	
	9.6%	(3.4–15.7%)	0.0784	
	5.6%	(−0.1–11.3%)	>0.999	
	3.5%	(−4.6–11.6%)	>0.999	
Occipital	−5.9%	(−9.2–−2.7%)	>0.999	
	−0.3%	(−3.3–2.7%)	>0.999	
	2.2%	(−0.4–4.8%)	>0.999	
	−0.3%	(−5.6–5%)	>0.999	
Calcarine cortex (Calc)	−4.7%	(−11.4–2%)	>0.999	
	−2.2%	(−8.7–4.3%)	>0.999	
	5.8%	(0.5–11.1%)	0.8568	
	−4%	(−15.2–7.1%)	>0.999	
Cuneus (Cun)	−9.3%	(−15.7–−2.8%)	>0.999	
	−2.4%	(−7.8–3%)	>0.999	
	0.7%	(−4.6–6%)	>0.999	
	−0.7%	(−9.6–8.2%)	>0.999	
Lingual gyrus (LiG)	−2.9%	(−8.2–2.4%)	>0.999	
	0.5%	(−4.3–5.3%)	>0.999	
	4.6%	(−0.4–9.5%)	>0.999	
	−0.1%	(−9.6–9.4%)	>0.999	
Fusiform gyrus occipital (OFuG)	−3.2%	(−11.9–5.4%)	>0.999	
	−3.1%	(−10.7–4.6%)	>0.999	
	7.5%	(0.2–14.7%)	>0.999	
	8.7%	(−5.1–22.6%)	>0.999	
Inferior occipital gyrus (IOG)	−6.2%	(−13–0.7%)	>0.999	
	−1%	(−7.4–5.5%)	>0.999	
	−4%	(−10–2%)	>0.999	
	−6.6%	(−16.5–3.3%)	>0.999	
Middle occipital gyrus (MOG)	−4.6%	(−11.8–2.7%)	>0.999	
	−1.1%	(−7–4.7%)	>0.999	
	3.2%	(−2.8–9.3%)	>0.999	
	−7.3%	(−18.8–4.2%)	>0.999	
Superior occipital gyrus (SOG)	−13.4%	(−23>1–−3.8%)	>0.999	
	0.5%	(−6.7–7.8%)	>0.999	
	−2%	(−8.8–4.8%)	>0.999	
	10.5%	(−3.3–24.2%)	>0.999	
Occipital pole (OCP)	−6.6%	(−14.7–1.4%)	>0.999	
	8.8%	(1–16.5%)	0.67	
	3.8%	(−2.7–10.3%)	>0.999	
	3.4%	(−8.6–15.4%)	>0.999	
Limbic	−1%	(−4.3–2.3%)	>0.999	
	−0.9%	(−3.8–2%)	>0.999	
	1.4%	(−1.4–4.1%)	>0.999	
	−1.8%	(−6.3–2.8%)	>0.999	
Entorhinal area (Ent)	−2.2%	(−9.3–4.9%)	>0.999	
	−9.3%	(−16.1–−2.4%)	>0.999	
	−3.2%	(−9.5–3.1%)	>0.999	
	10.4%	(2.2–18.6%)	0.4608	
Anterior cingulate gyrus (ACgG)	1.7%	(−6–9.4%)	>0.999	
	6.5%	(−0.3–13.2%)	>0.999	
	8.2%	(1.2–15.3%)	0.621	
	−6.1%	(−17.7–5.5%)	>0.999	
Middle cingulate gyrus (MCgG)	0.5%	(−4.7–5.7%)	>0.999	
	1.3%	(−2.8–5.4%)	>0.999	
	2.9%	(−1.8–7.5%)	>0.999	
	−2.9%	(−10.8–5.1%)	>0.999	
Posterior cingulate gyrus (PCgG)	−1.9%	(−6>2–2.3%)	>0.999	
	−2.1%	(−6–1.8%)	>0.999	
	−5.6%	(−9.2–−2.1%)	>0.999	
	0.4%	(−5.5–6.3%)	>0.999	
Parahippo-campal gyrus (PHG)	−6%	(−10.8–−1.1%)	>0.999	
	−9.4%	(−14.6–−4.2%)	>0.999	
	0.9%	(−3.9–5.7%)	>0.999	
	−6%	(−14.7–2.8%)	>0.999	
Insular	8.1%	(5–11.2%)	0.0064**	0.59
	9.9%	(5.4–14.3%)	0.0064**	0.524
	2.9%	(0.4–5.4%)	0.621	
	7.2%	(1.8–12.6%)	0.32	
Anterior insula (AIns)	9.6%	(5.9–13.3%)	0.0064**	0.59
	9.1%	(5.2–13%)	0.0064**	0.524
	3.8%	(0.2–7.4%)	0.9653	
	7%	(0.5–13.4%)	>0.999	
Posterior Insula (Pins)	12.9%	(8.1–17.7%)	0.0064**	0.59
	8.4%	(3–13.8%)	0.0754	
	3.6%	(0.2–7%)	0.9653	
	6%	(−1.1–13.1%)	>0.999	
Central operculum (CO)	4.5%	(−0.9–9.8%)	>0.999	
	11.7%	(6–17.4%)	0.0064**	0.524
	1.8%	(−2.6–6.2%)	>0.999	
	10.9%	(2.8–18.9%)	0.3072	
Frontal operculum (FO)	11.7%	(3.5–19.9%)	0.1484	
	11.3%	(2.5–20.1%)	0.3276	
	5%	(−3–13.1%)	>0.999	
	5.6%	(−8.2–19.4%)	>0.999	
Parietal operculum (PO)	3.7%	(−5.2–12.7%)	>0.999	
	8.4%	(−1.1–17.9%)	>0.999	
	1%	(−6.9–8.9%)	>0.999	
	3.6%	(−9.7–16.9%)	>0.999	

^*^ *P* < 0.05.

^**^ *P* < 0.01.

The hypothesis that the reduction in supratentorial parenchymal brain volumes is a correlate of atrophy, would also result in an expansion of external CSF spaces (exCSF). Consequently, the CSF spaces were also examined. Correspondingly, these volume differences behaved reciprocal: Patients with biMMD showed significant volume difference in CSF spaces compared to the same age cohort of HC. This was observed in both the total CSF volume and in the subregions of the exCSF or the internal CSF (inCSF) spaces of the supratentorial ventricular system. Relevant volume differences could not be detected for the fourth ventricle (Fig. 3A). For biMMD in the age groups 30–45 and 46–60 years, there was a significant increase in the mean volume of the exCSF spaces by 26.4% (95% CI 17.0–35.9%; *P* < 0.001; *d* = 1.29) and by 28.4% (95% CI 17.1–39.7%; *P* < 0.001; *d* = 1.38), respectively.

### Volumetric comparison of the MMD and HC cohorts–infratentorial

As MMD is typically regarded as a disease affecting the proximal supratentorial circulation, infratentorial ROIs were studied as a control. Surprisingly, significant volume differences of infratentorial structures were also observed compared to HC. In particular with increasing age, there was a trend towards significant cerebellar volume decrease, with cerebellar WM (cerWM) being more affected than cerebellar GM (cerGM). In the biMMD cohort of 46–60 years, the decrease of cerWM was most pronounced compared to the HC group, with a volume reduction of 11.6% (95% CI 3.7–19.5%; *P* = 0.0025, *d* = 0.87). Furthermore, a significant reduction of brainstem volume in patients aged 46–60 [reduction of 8.5% (95% CI 3.5–13.5%; *P* = 0.0006, *d* = 0.96)] and >60 years was also remarkable and will be addressed further in the discussion (Fig. 3C).

### Volumetric analysis in uniMMD and in MMD without ischaemia

**Figure 4:**
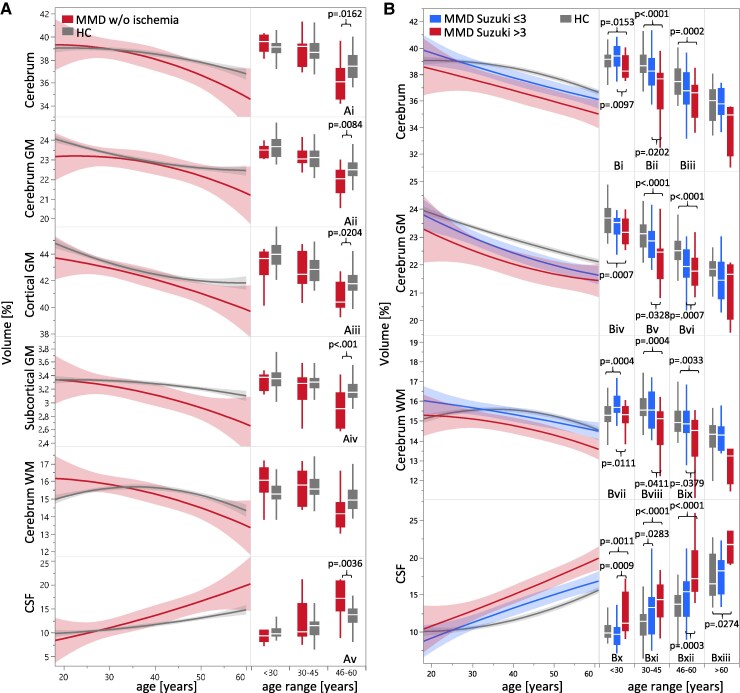
**Differences in brain volume in MMD without ischaemia and with the consideration of Suzuki grade.** (**A**) Comparison of MMD patients without ischaemia (red) with HC (selected ROIs). Right side: Boxplots of ROI volumes split into age groups <30 (*n* = 105), 30–45 (*n* = 77) and 46–60 years (*n* = 70). The *t* statistics of the *t*-tests performed are Ai: *t*(68) = 2.88, Aii: *t*(68) = 3.09, Aiii: *t*(68) = 2.79, Aiv: *t*(68) = 3.96, Av: *t*(68)=−3.39. *P*-values adjusted according to Bonferroni–Holm correction across all six examined ROIs per age group (6 × 3). Note: The study population aged >60 years included only two patients with MMD without ischaemia. Therefore, a graphical and statistical analysis was not performed. (**B**) All patients with MMD divided into Suzuki minor (≤3, green) and major grades (<3, red) compared with HC. Right side: Boxplots of ROI volumes split into age groups <30 (*n* = 119), 30–45 (*n* = 103), 46–60 (*n* = 100) and >60 years (*n* = 48). ANOVA test results are Bi: *F*(2118) = 4.86, Bii: *F*(2102) = 12.10, Biii: *F*(2.99) = 9.12, Biv: *F*(2118) = 7.68, Bv: *F*(2102) = 13.06, Bvi: *F*(2.99) = 13.66, Bvii: *F*(2118) = 4.78, Bviii: *F*(2102) = 7.88, Bix: *F*(2.99) = 5.64, Bx: *F*(2118) = 7.08, Bxi: *F*(2102) = 10.90, Bxii: *F*(2.99) = 21.37, Bxiii: F(2.47) = 3.60. Tukey-Kramer *post hoc P*-values of significance are shown for the respective pairs of bars.

In uniMMD without ischaemic lesions in the affected hemisphere (*n* = 13) intraindividual comparison was performed with the non-affected hemisphere: cerebral volume of the affected hemisphere was significantly reduced by 1.4% (95% CI 0.5–2.2%; *P* = 0.0020; *d_z_* = 0.98), the proportions of WM by 1.7% (95% CI 0.4–2.9%; *P* = 0.0081, *d_z_* = 0.77) and those of GM by 1.2% (95% CI 0.5–1.9%; *P* = 0.0010; *d_z_* = 1.08). Age-controlled WBV of all MMD patients without ischaemic lesions (*n* = 18) revealed significant loss of brain volume across almost all examined macrostructures of GM, WM and increase of CSF spaces compared to HC at older ages between 46 and 60 years (Fig. 4A).

### Volumetric analysis in MMD correlated to the stage of the disease

**Figure 5:**
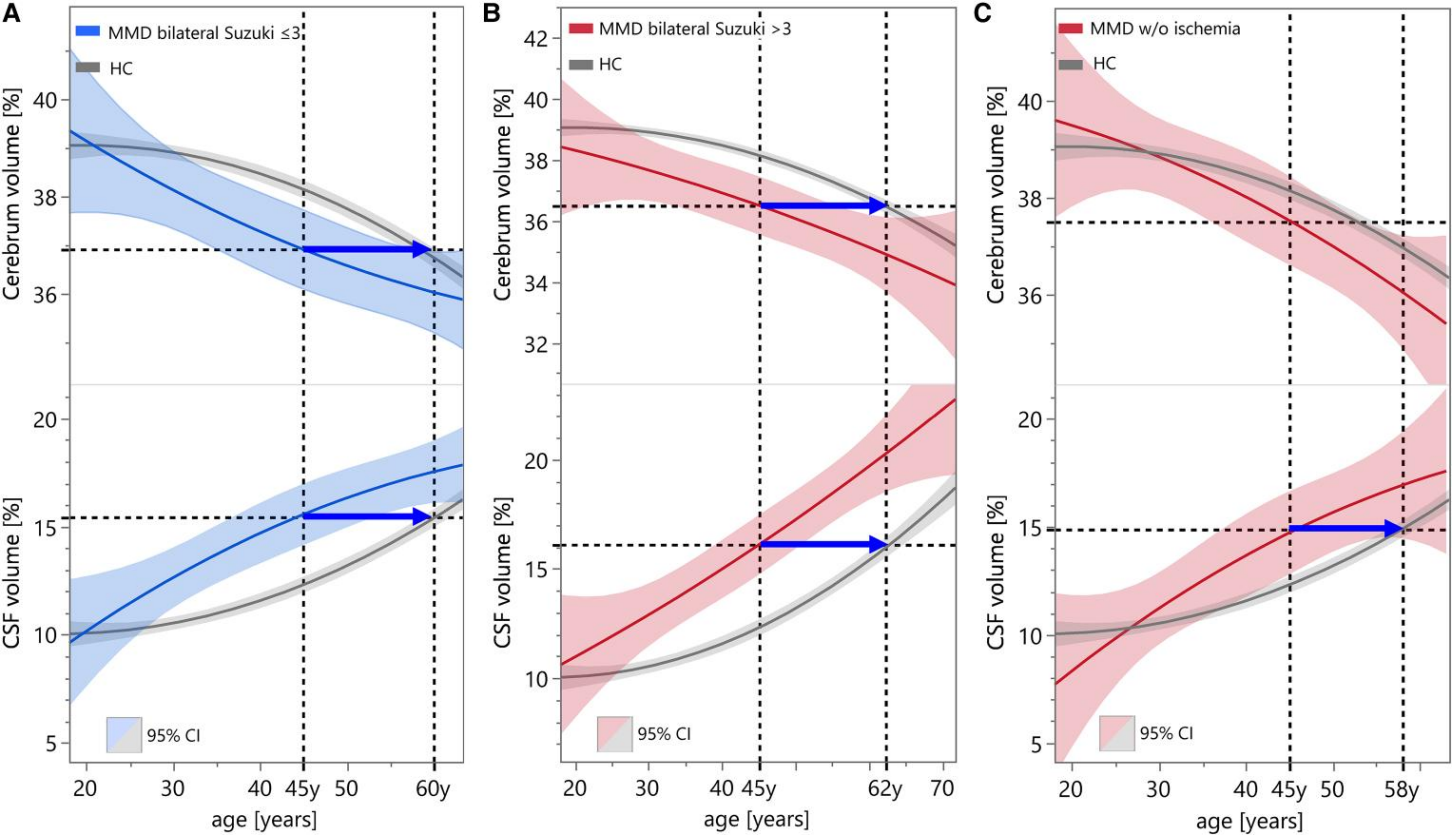
**Hypothetical age shift for a 45-year-old MMD patient.** Hypothetical age shift (blue arrows) for a 45-year-old MMD patient with corresponding ROI volumes in HC. (**A**) biMMD with Suzuki grade ≤3 (green). (**B**) biMMD with Suzuki grade >3 (red). (**C**) MMD without ischaemia.

When analysing the WBV results regarding in relation to the disease stage, no significant discriminatory power was found with regard to the Suzuki classification.^2^ Only the subdivision into minor (Suzuki ≤ 3) and major (Suzuki > 3) resulted in significant volume differences in the ROIs examined (Fig. 4B). As shown by the arrows, WBV of a 45-year-old MMD patient without infarctions would correspond to the brain of a 57.4-year-old healthy person. In biMMD at 45 years and minor Suzuki grades, the cerebrum and CSF space correspond to those of a healthy person of 58.6 and 60.7 years, respectively, and in major Suzuki grades to those of a healthy person aged 62.1 years (Fig. 5A–C).

## Discussion

MMD leads to impaired haemodynamics caused by steno-occlusive changes of the basal cerebral arteries, which can be assessed by functional imaging using SPECT, PET–CT and fMRI.^11,12^ When compensatory mechanisms become insufficient, transient attacks or even manifest ischaemic lesions may occur.^13,14^ In addition to clinically well detectable sensorimotor symptoms, neurocognitive deficits can often be found, but are difficult to measure and require specific neuropsychological testing.^3,4,15-18^ Evidence increases that this also applies to patients without manifest ischaemic lesions depicted on MRI.^16,17,19^ Such silent cognitive decline might occur even before evident clinical symptoms.^19^ Some measurable effects of chronic low-flow hypoperfusion of MMD patients’ brains have been described previously, but not with a specific focus on quantification by high-resolution volumetric and in comparison to healthy controls.^20^ In this study, we have compared a large cohort of surgically untreated MMD patients to an age-adjusted healthy control population regarding brain volume, using available high-resolution MRI. We have found significant volume differences in the investigated ROIs and macrostructures across almost all age groups and specifically also in MMD patients without macroscopically depictable infarctions and with increasing age. This might underline the hypothesis of brain atrophy as a sequela of chronic hypoperfusion.^21^ Especially frontal and insular ROIs showed a significant volume decline in our analysis (Tables 2 and 3). This possibly highlights their role as typical vascular territories affected by MMD, which are usually treated by superior temporal artery, middle cerebral artery (STA-MCA) and anterior cerebral artery (STA-ACA) bypasses in tailored revascularization procedures. Other studies have already examined GM differences by measuring cortical thickness in paediatric MMD patients compared to healthy controls: as a result, abnormal cortical representation of the insula, caudate, postcentral, precuneus and cingulate regions were shown.^22^ In adult MMD patients such findings are also described, but the distribution of thickened and thinned cGM was more heterogeneous and this was suggested as a potential marker for disease severity.^23^ Accordingly, regionally reduced cortical thickness was also seen in correlation with haemodynamic impairment seen in PET/CT with a higher regional oxygen extraction fraction ratio.^24^ Yet, it is important to distinguish between GM and WM in WBV, since WM has only about a quarter of the capillary density and cerebral blood flow (CBF) compared to GM.^25^ At least in animal models, chronic cerebral hypoperfusion has been shown to cause myelin damage that precedes WM axon damage.^21^

In an earlier study, diffusion-weighted MRI (DW-MRI) was used to describe demyelination changes, myelin sheath diameter and WM maturation in MMD as indicators for the early prediction of chronic damage in WM.^26^ This chronic and ongoing damage in the microstructure of WM has been shown to occur primarily in watershed regions, even in the absence of manifest strokes in paediatric MMD.^27^

In adult MMD patients, more deviations in WM diffusion scores than in GM have already been described, which might be linked to the development of cognitive dysfunction.^28^ Speaking of such cognitive decline in MMD patients without stroke or haemorrhage, CBF impairment and disrupted brain network connections may be important causes of this cognitive function loss and may even precede manifest clinical symptoms.^19^ Recent studies showed that WM hyperintensities (WMH) appear to correlate with global cognition, memory, semantic memory and executive function.^29,30^ These studies also described periventricular WMH in particular as a possible marker of ischaemic vulnerability in patients with MMD.^29,30^

In addition to the direct consequences of chronic hypoperfusion, its indirect consequences must also be addressed: secondary neurodegeneration might be based on a pathomechanism similar to that described for MCA infarction. Not only is there ischaemia in the affected vascular territory, but also pathological changes with neuronal cell death, axonal degeneration and gliosis in distant non-ischaemic regions that maintain synaptic connections with the affected area, such as the basal ganglia, thalamus, brainstem or cerebellum.^31,32^

Regarding such indirectly affected areas in MMD, a recently published case–control study dealing with a patient population similar in size to the present one, but exclusively considering scGM, came to comparable results: extensive structural deficits were found in the thalamus, caudate, putamen, hippocampus, amygdala, pallidum and nucleus accumbens, which correlated with disease duration and intracranial arterial changes and were probably due to chronic haemodynamic insufficiency.^33^ This supports our results, as scGM particularly of the caudate, pallidum and putamen, were also reduced in our MMD cohort, representing a possible correlate of delayed secondary neurodegeneration. The volume deficits in the subgroup analysis of non-ischaemic MMD patients at an older age seem to support this hypothesis. Additionally, we also saw significant infratentorial volume differences of the cerebellum and brainstem, although its direct blood supply from the vertebral and basilar arteries is generally not considered to be affected by MMD. It might be possible that these volume differences are consequences of secondary neurodegeneration due to the decline of cortico-ponto-cerebellar fibre tracts. Further evidence for indirect cerebellar involvement in MMD regarding cerWM was also found in a small comparative study.^34^ MMD may therefore be understood not only as a local disease of single or multiple vascular territories, but also as a global cerebrovascular disease of the entire brain because of indirect damage by secondary neurodegeneration.

## Limitations

This is a retrospective study with the risk of selection bias. It is important to note that this is a cross-sectional study, and thus the disease onset of MMD and duration are unknown in the vast majority of cases. However, it must be assumed that this has an influence on the WBV. Additionally, technical issues might lead to variability in brain volume during the acquisition of MRI. There is a risk that the automated volumetric evaluation of the MR images might have led to random variations of the volumes calculated. However, the safety measures applied for this study indicate a high likelihood of reliable results from our evaluation pipeline throughout the affected patients and healthy controls. Further, 15 hemispheres were excluded due to failure of automatic segmentation, most likely caused by large-sized infarctions.

## Conclusion

This study quantifies brain atrophy in a large group of MMD patients with and without infarctions seen on MRI, adjusted for age and disease stage with respect to different cerebral ROIs. This highlights the direct impact of the disease on affected vascular territories, as well as the indirect consequences through secondary neurodegeneration on the brain as a whole. These findings provide a deeper understanding of the burden of MMD, emphasizing the importance of timely diagnostics and tailored revascularization for patients.

## Data Availability

The data that support the findings of this study are available from the corresponding author, upon reasonable request.
